# A determination of $\alpha _s(m_Z)$ at ${{\textrm{aN}}}^3{{\textrm{LO}}}_{{\textrm{QCD}}}\otimes {{\textrm{NLO}}}_{{\textrm{QED}}}$ accuracy from a global PDF analysis

**DOI:** 10.1140/epjc/s10052-025-14676-y

**Published:** 2025-09-16

**Authors:** NNPDF Collaboration, Richard D. Ball, Andrea Barontini, Juan Cruz-Martinez, Stefano Forte, Felix Hekhorn, Emanuele R. Nocera, Juan Rojo, Roy Stegeman

**Affiliations:** 1 The Higgs Centre for Theoretical Physics, University of Edinburgh, JCMB, KB, Mayfield Rd, Edinburgh, EH9 3JZ Scotland; 2 Tif Lab, Dipartimento di Fisica, Università di Milano and INFN, Sezione di Milano, Via Celoria 16, 20133 Milan, Italy; 3 Theoretical Physics Department, CERN, 1211 Geneva 23, Switzerland; 4 Department of Physics, University of Jyvaskyla, P.O. Box 35, 40014 Jyvaskyla, Finland; 5 Helsinki Institute of Physics, University of Helsinki, P.O. Box 64, 00014 Helsinki, Finland; 6 Dipartimento di Fisica and INFN, Sezione di Torino, Università degli Studi di Torino, Via Pietro Giuria 1, 10125 Turin, Italy; 7 Department of Physics and Astronomy, Vrije Universiteit, 1081 HV Amsterdam, The Netherlands; 8 Nikhef Theory Group, Science Park, 105, 1098 XG Amsterdam, The Netherlands

## Abstract

We present a determination of the strong coupling $\alpha _s(m_Z)$ from a global dataset including both fixed-target and collider data from deep-inelastic scattering and a variety of hadronic processes, with a simultaneous determination of parton distribution functions (PDFs) based on the NNPDF4.0 methodology. This determination is performed at NNLO and approximate ${\textrm{N}}^3$LO (${\textrm{aN}}^3$LO) perturbative QCD accuracy, including QED corrections and a photon PDF up to NLO accuracy. We extract $\alpha _s$ using two independent methodologies, both of which take into account the cross-correlation between $\alpha _s$ and the PDFs. The two methodologies are validated by closure tests that allow us to detect and remove or correct for several sources of bias, and lead to mutually consistent results. We account for all correlated experimental uncertainties, as well as correlated theoretical uncertainties related to missing higher order perturbative corrections (MHOUs). We study the perturbative convergence of our results and the impact of QED corrections. We assess individual sources of uncertainty, specifically MHOUs and the value of the top quark mass. We provide a detailed appraisal of methodological choices, including the choice of input dataset, the form of solution of evolution equation, the treatment of the experimental covariance matrix, and the details of Monte Carlo data generation. We find $\alpha _s(m_Z)=0.1194^{+0.0007}_{-0.0014}$ at ${\textrm{aN}}^3$ ${\textrm{LO}}_{\textrm{QCD}}\otimes {\textrm{NLO}}_{\textrm{QED}}$ accuracy, consistent with the latest PDG average and with recent lattice results.

## Introduction

The precise knowledge of the strong coupling $\alpha _s$ is one of the main bottlenecks towards reaching percent or sub-percent accuracy in the computation of hadron collider processes [1]. Conversely, many of the most accurate determinations of the strong coupling are obtained from processes that involve hadrons in the initial state [2, 3]. These determinations inevitably involve knowledge of hadron structure, as encoded in parton distribution functions (PDFs) [4, 5], and it has now been known for some time [6] that reliable unbiased results can only be obtained if $\alpha _s$ and the PDFs are simultaneously determined, as opposed to using a fixed PDF set.

Extractions of $\alpha _s(m_Z)$ together with the PDFs have been carried out by various groups over the years, with the most precise recent results obtained by groups that make use of a global dataset involving several disparate pieces of experimental information [7–10]. These PDF-based determinations differ in the input dataset, the accuracy of the theory calculations, and the fitting methodology. In particular, the NNPDF collaboration has presented several determinations at NNLO QCD accuracy, based not only on increasingly wider datasets and more refined PDF determination methodology, but also on a more sophisticated treatment of the correlation between $\alpha _s$ and the PDFs.

Specifically, in Ref. [11] a first result based on the NNPDF2.1 [12, 13] methodology was obtained, by repeating the PDF determination for several fixed values of $\alpha _s$ and extracting $\alpha _s$ and its uncertainty from the $\chi ^2(\alpha _s)$ parabolic profile. While this gives the correct central value, it generally underestimates the uncertainty because the correlation between $\alpha _s$ and the PDFs is not fully accounted for. Indeed, for an accurate determination of the uncertainty, knowledge of the $\chi ^2$ paraboloid in joint $\alpha _s$ and PDF space is necessary. This in turn requires the simultaneous determination of $\alpha _s$ and the PDFs, as opposed to the determination of PDFs for different fixed values of $\alpha _s.$ This result was accomplished in Ref. [8], based on NNPDF3.1 [14] methodology, through a correlated replica method (CRM), that involves performing a family of PDF replica determinations using different $\alpha _s$ values to each individual Monte Carlo data replica.

Since then, progress has been made in various directions: the PDF determination methodology, theory treatment and uncertainty treatment; the $\alpha _s$ extraction methodology; and the validation methodology. Concerning the PDF determination, the NNPDF3.1 methodology has been superseded by the more precise and accurate NNPDF4.0 [15, 16] methodology, based on modern machine learning techniques. On the theory side, thanks to recent progress on the ${\textrm{N}}^3$LO calculations of splitting functions (see [17–19] for the latest results) it is now possible to determine PDFs at approximate ${\textrm{N}}^3$LO (${\textrm{aN}}^3$LO) accuracy, as done by MSHT [20] and NNPDF [21]. Moreover, it is now clear that the inclusion of the photon PDF in joint QCD$\otimes$QED evolution equations is necessary for percent accuracy, and both MSHT [22] and NNPDF [23, 24] have included QED effects in their ${\textrm{aN}}^3$LO PDF determinations (see also [25] for their combination).

As far as uncertainties are concerned, it is now recognized that in order to obtain accurate PDF uncertainties it is necessary to include correlated missing higher order uncertainties (MHOUs) on the theory predictions for the processes used in the PDF determination. This was done by MSHT at ${\textrm{aN}}^3$LO [20] using a nuisance parameter formalism, and by NNPDF both at NNLO [26] and ${\textrm{aN}}^3$LO [21] using the theory covariance matrix formalism developed in Refs. [27–31] to account for MHOUs (as well as nuclear uncertainties).

As for the $\alpha _s$ extraction method, it was shown in Ref. [32] that the value and uncertainty on any theory parameter, such as the parameters that determine the shape of the PDFs or indeed $\alpha _s,$ can be determined by Bayesian inference from knowledge of the covariance matrix of theory parameters. When applied to $\alpha _s$ this theory covariance method (TCM) provides an alternative way of extracting the strong coupling that also fully keeps into account the correlation to PDFs. Finally, it is now recognized that closure tests [15, 33–36] are necessary for a full validation of the methodology used to determine PDF uncertainties, and thus also $\alpha _s.$

We present here a new determination of $\alpha _s$ that includes all these developments. Specifically, we determine $\alpha _s$ and PDFs based on NNPDF4.0 methodology, using theory up to ${\textrm{aN}}^3$LO QCD, with QCD$\otimes$QED including a photon PDF up to NLO, with full inclusion of MHOUs. Results are obtained using both the CRM and TCM. These methodologies are validated by a closure test that allows us to detect sources of bias: specifically, those related to the treatment of multiplicative uncertainties and to positivity constraints. We show that the two methodologies lead to consistent results, and we appraise the impact of correlations between PDFs and $\alpha _s$ and MHOUs. We assess perturbative convergence and the effect of QED corrections and we study the impact of positivity. We check the stability of our result upon a sizable number of methodological and parametric variations, including the form of the solution of evolution equations, the value of the top quark mass, and the Monte Carlo data generation. We also study dataset dependence and specifically show agreement with our previous $\alpha _s$ value of Ref. [15] if the same dataset is adopted.

The paper is organized as follows. In Sect. 2 we review the methods that we use for our $\alpha _s$ determination: the CRM of Ref. [8], and the TCM of Ref. [32] and its specific application to $\alpha _s.$ These two methodologies are validated by means of closure tests in Sect. 3, where we detect and characterize possible sources of bias. Our main results for $\alpha _s(m_Z)$ are presented in Sect. 4, where we study their stability with respect to variations of theory, methodology, and experimental input. Conclusions are drawn in Sect. 5, where we also provide information on how to access our results (specifically the LHAPDF grids [37]), all of which are made public.

## Methodologies for $\alpha _s(m_Z)$ extraction

We perform a simultaneous determination of $\alpha _s$ and PDFs using two different methodologies that fully take into account the correlations between $\alpha _s$ and the PDFs. The first is the correlated replica method (CRM), used for the extraction of $\alpha _s$ in Ref. [8]. It is based on a maximum likelihood estimate of all parameters which relies on frequentist Monte Carlo resampling. The second is based on the theory covariance method (TCM), first introduced in Ref. [32] as a means to account for correlations between MHOUs on theory predictions obtained from a given PDF set, and MHOUs on the predictions that had been used to determine them. The TCM, applied for the first time to the determination of $\alpha _s$ in the present, relies on Bayesian posterior parameter estimation. The Monte Carlo method and the Bayesian method are statistically equivalent for linear error propagation of Gaussian uncertainties [38], and below we will explicitly check that indeed the TCM and CRM lead to consistent results both in a closure test and with real data. It would be in principle also possible to determine $\alpha _s$ and the PDFs simultaneously using the SIMUnet method [39, 40], which involves interpolating the theory for given PDF input as $\alpha _s$ is varied, and then treating $\alpha _s$ as a parameter in the numerical optimization; however, we do not use this technique in this paper.

### The correlated replica method

For completeness and in order to set up the notation, we provide a self-contained introduction to the CRM and a brief summary of the NNPDF methodology on which it is based, referring the reader to Ref. [8] for more details.

The Monte Carlo inference method adopted by NNPDF is based on starting from a Monte Carlo representation of the probability distribution of the original experimental data, and determining for each data replica an optimal PDF represented by a neural network, found through conditional optimization of a suitable loss function, thus obtaining a Monte Carlo representation of the probability distribution of PDF replicas. The data replicas $\{ D_i^{(k)}\},$ where *i* denotes the data points $i\in \{1,\ldots ,N_{\textrm{dat}}\}$ and *k* the replica numbers $k\in \{1,\ldots ,N_{\textrm{rep}}\},$ are obtained by sampling the original data from a multi-Gaussian distribution such that

2.1 $$\begin{aligned} \lim _{N_{\textrm{rep}}\rightarrow \infty } {\textrm{cov}}\left( D_i^{(k)}, D_j^{(k)}\right) =C_{ij} , \end{aligned}$$

where *C* is the total covariance matrix of the data, in turn given by a sum of contributions of an experimental $t_0$ covariance matrix $C^{\textrm{exp}}_{t_0}$ [41, 42] and a theory covariance matrix $C^{\textrm{th}}$ that includes MHOUs and other theory uncertainties (such as nuclear uncertainties) [27–31]:

2.2 $$\begin{aligned} C_{ij} = C^{\textrm{exp}}_{t_0,ij}+C^{\textrm{th}}_{ij} . \end{aligned}$$

An optimal PDF replica, characterized by parameters ${\theta }^{(k)},$ is then determined for each data replica by minimizing a loss function computed on a training subset of data and stopping the training conditionally on the loss computed on the remaining (validation) data subset. For the theoretical prediction of the *i*-th data point evaluated with the *k*-th PDF replica, denoted $T_{i}({\theta }^{(k)},\alpha _s),$ the loss function is

2.3 $$\begin{aligned} E^{(k)}\left( {\theta }^{(k)},\alpha _s\right) =  &   \frac{1}{ N_{\textrm{dat}}} (T({\theta }^{(k)},\alpha _s) - D^{(k)})^T \nonumber \\  &   C^{-1} \, (T({\theta }^{(k)},\alpha _s) - D^{(k)} ),\nonumber \\ \end{aligned}$$

where we have adopted a vector notation so that *T* and *D* are $N_{\textrm{dat}}$-component vectors, $T^T$ and $D^T$ the corresponding transpose vectors, and *C* is an $N_{\textrm{dat}}\times N_{\textrm{dat}}$ real symmetric matrix. All indices $i,j\in \{1,\ldots ,N_{\textrm{dat}}\}$ are then implicitly summed over, but the replica number *k* and $\alpha _s$ dependence are left explicit. Note that in the definition of Eq. (2.3) the theoretical predictions, and thus the dependence on $\alpha _s,$ enter both directly, as displayed, but also indirectly when constructing the $t_0$ covariance matrix $C^{\textrm{exp}}_{t_0}$ and the theory covariance matrix $C^{\textrm{th}}.$ As we will demonstrate in Sect. 3, this dependence of the covariance matrix on $\alpha _s$ may lead to biased results if not treated with care.

In the CRM, the PDFs parameters ${\theta }^{(k)}$ are determined for each fixed replica *k*,  for a number of different fixed values of $\alpha _s,$ thereby leading to an $\alpha _s$ dependent vector of optimized parameters for each replica, that we denote $\overline{\theta }^{(k)}(\alpha _s).$ A maximum likelihood estimate of $\alpha _s$ for each data replica may then be obtained as

2.4 $$\begin{aligned} \alpha _s^{(k)} = \operatorname {arg min}\left[ E^{(k)}\left( \overline{\theta }^{(k)}(\alpha _s),\alpha _s\right) \right] . \end{aligned}$$

A continuous function $E^{(k)}\left( \overline{\theta }^{(k)}(\alpha _s),\alpha _s\right)$ of $\alpha _s$ may be obtained by interpolating the values of the loss Eq. (2.3) obtained with all the given values of $\alpha _s,$ which in practice means fitting them to a parabola, or possibly a higher order polynomial. This then leads to a Monte Carlo representation of the probability distribution in the joint $(\alpha _s,$ PDF) space, whence the most likely value of $\alpha _s$ and associated confidence levels (CL) can be determined, as well as the correlations with the PDFs. We refer to Ref. [8] for technical details on the implementation of the CRM in the NNPDF framework, which we follow in this work.

It is important to observe that the CRM provides a more reliable estimate of the $\alpha _s$ uncertainty than that which is obtained by neglecting the correlation between $\alpha _s$ and the PDF, as was the case in the earlier NNPDF2.1-based determination of $\alpha _s$ [11, 43]. In this simpler procedure (sometimes still used today) the central theory prediction

2.5 $$\begin{aligned} T^{(0)}(\alpha _s) = \frac{1}{N_{\textrm{rep}}} \sum _{k=1}^{N_{\textrm{rep}}} T\left( \overline{\theta }^{(k)}(\alpha _s),\alpha _s\right) , \end{aligned}$$

is used to evaluate the loss

2.6 $$\begin{aligned} \chi ^2(\alpha _s) = ( T^{(0)}(\alpha _s) - D)^T \,C^{-1} \, ( T^{(0)}(\alpha _s) - D ). \end{aligned}$$

The best fit value of $\alpha _s$ is then

2.7 $$\begin{aligned} \alpha _s^{(\mathrm min)} = \operatorname {arg min}\left[ \chi ^{2}(\alpha _s)\right] , \end{aligned}$$

with 68% CL uncertainty found from the $\Delta \chi ^2 = 1$ range about the best-fit value. This leads in general to an underestimate of the uncertainty on $\alpha _s,$ as demonstrated in Ref. [8], since it does not account for the correlation in uncertainty between $\alpha _s$ and the PDF.

### The theory covariance method

The TCM was originally introduced in Ref. [32] as a means to avoid double-counting of theory uncertainties when computing predictions for a process that is correlated with data whose theoretical uncertainties have been included in the PDF determination. However, the same technique can also be used to obtain a Bayesian determination of the maximum likelihood value of any nuisance parameter. Performing this determination for each data replica leads to a determination of the probability distribution of the nuisance parameter. Hence, by viewing the deviation of the value of $\alpha _s$ (or indeed any other parameter entering the theory predictions) from its prior as a nuisance parameter, the method can be used to obtain a determination of $\alpha _s$ and its associated probability distribution. Here, we first briefly summarize the general method, then describe its application to the determination of $\alpha _s.$

The starting observation is the well-known result that any correlated Gaussian uncertainty can be represented as a shift of either the theory or the data through a nuisance parameter $\lambda$: $T \rightarrow T+\lambda \beta$ for a theory uncertainty, or $D \rightarrow D-\lambda \beta$ for a data uncertainty. Henceforth we will assume for definiteness that the correlated uncertainty is a theory uncertainty (hence the choice of sign in the definition of $\lambda$) though the treatment is entirely symmetric under the interchange of *T* and *D*. Such a correlated shift of the theory predictions is equivalent to adding a contribution

2.8 $$\begin{aligned} S_{ij}=\beta _i\beta _j , \end{aligned}$$

to the covariance matrix $C_{ij},$ Eq. (2.2).

In a Bayesian framework, this can be easily proven as follows. The probability *P*(*T*|*D*) can be obtained by marginalizing over $\lambda$ the joint probability $P(T|D,\lambda )$ multiplied by the prior $P(\lambda )$:

2.9 $$\begin{aligned} P(T|D)=\int d\lambda P(T|D,\lambda )P(\lambda ). \end{aligned}$$

For Gaussian distributed observables

2.10 $$\begin{aligned} P(T|D,\lambda ) \propto \exp \left[ -\frac{1}{2} (T + \lambda \beta - D)^T C^{-1} \, (T+\lambda \beta - D) \right] ,\nonumber \\ \end{aligned}$$

so, if the prior $P(\lambda )$ is a univariate Gaussian centered at zero

2.11 $$\begin{aligned} P(\lambda )\propto \exp \left( -\frac{\lambda ^2}{2}\right) , \end{aligned}$$

then it is straightforward to perform the integral over $\lambda$ by completing the square, to give

2.12 $$\begin{aligned} P(T|D) \propto \exp \left[ -\frac{1}{2} (T- D)^T (C+S)^{-1} \, (T- D) \right] .\nonumber \\ \end{aligned}$$

Thus the correlated uncertainty parametrized by the nuisance parameter $\lambda$ may be incorporated simply by adding the contribution *S*,  given by Eq. (2.8), to the original covariance matrix *C*,  Eq. (2.2).

The advantage of this point of view is that Bayes’ theorem also determines the posterior distribution of the nuisance parameter:

2.13 $$\begin{aligned} P(\lambda | T,D) \propto \exp \left( -\frac{1}{2}Z^{-1}\left( \lambda - \bar{\lambda }(T,D)\right) ^2\right) , \end{aligned}$$

which is a Gaussian of width *Z* centered at $\bar{\lambda },$ with

2.14 $$\begin{aligned} \bar{\lambda }(T,D)&=\beta ^T (C+S)^{-1} (D-T) , \end{aligned}$$

2.15 $$\begin{aligned} Z&= 1-\beta ^T (C+S)^{-1} \beta . \end{aligned}$$

This way of treating correlated uncertainties can be integrated within the NNPDF methodology by simply representing any of the correlated uncertainties included in the covariance matrix through a nuisance parameter. Assuming for the sake of argument that we start from some covariance matrix *C*,  Eq. (2.2), and we want to add to *C* a new correlated theory uncertainty, represented by a covariance matrix *S*,  and uncorrelated to any of the uncertainties already included in *C*,  we perform determinations of $\bar{\lambda }$ by generating Monte Carlo data replicas as in Eq. (2.1), then fit them using the loss function Eq. (2.3), but in each case with the covariance matrix *C* replaced by $C+S.$ In this way for each data replica $D^{(k)}$ we obtain associated PDF parameters $\overline{\theta }^{(k)},$ and thus theoretical predictions $T^{(k)},$ which we can use to determine an ensemble of replicas $\bar{\lambda }^{(k)}$ of the nuisance parameters, with expectation value

2.16 $$\begin{aligned} \bar{\lambda }^{(0)} = \beta ^T (C+S)^{-1}(D- T^{(0)}) , \end{aligned}$$

where $T^{(0)}$ is the central prediction, Eq. (2.5), and *D* the central data point. The variance of the nuisance parameter over the replica sample can also be computed analytically:

2.17 $$\begin{aligned} \bar{Z}\equiv 1 - \beta ^T (C+S)^{-1}\beta +\beta ^T (C+S)^{-1} X (C+S)^{-1}\beta ,\nonumber \\ \end{aligned}$$

where *X* is the covariance matrix of the theoretical predictions, averaged over the $N_{\textrm{rep}}$ PDF replicas:

2.18 $$\begin{aligned} X_{ij}= \frac{1}{N_{\textrm{rep}}}\sum _{k=1}^{N_{\textrm{rep}}}( T_i^{(k)} - T_i^{(0)})(T_j^{(k)} - T_j^{(0)}) . \end{aligned}$$

The whole derivation can be straightforwardly extended to the case of multiple nuisance parameters and we refer to Ref. [32] for further details.

We may apply this procedure to the determination of $\alpha _s(m_Z)$ by simply viewing the deviation of $\alpha _s,$ that was hitherto kept fixed at $\alpha _s(m_Z)=\alpha _s^{0},$ as a nuisance parameter. The value $\alpha _s^{0}$ is then viewed as a prior, the nuisance parameter is

2.19 $$\begin{aligned} \lambda = \alpha _s - \alpha _s^{0}, \end{aligned}$$

and the posterior distribution and uncertainty of $\lambda$ are determined from the data. Taking $\alpha _s^\pm$ either side of this central value to establish a prior uncertainty, the nuisance parameter is endowed with a Gaussian prior centered on zero, with width $\Delta \alpha _s^\pm = \alpha _s^\pm - \alpha _s^{0}.$ We assume a symmetric interval, so $|\Delta \alpha _s^+|=|\Delta \alpha _s^-|=\Delta \alpha _s.$ The value of $\Delta \alpha _s$ fixes the width of the prior, which should be chosen wide enough that final results are independent of the prior, though not so wide that one can no longer use standard linear error propagation.

The vector of prior widths of theory predictions $\beta$ is determined by linearizing the dependence of the central theoretical predictions $T^{(0)}(\alpha _s),$ Eq. (2.5), around $\alpha _s^{0}$:

2.20 $$\begin{aligned}  &   T^{(0)}\left( \ \alpha _s\right) = T^{(0)}(\alpha _s^{0}) + (\alpha _s - \alpha _s^{0}) \frac{\partial T^{(0)}\left( \alpha _s\right) }{\partial \alpha _s}\Bigg |_{\alpha _s=\alpha _s^{0}}\nonumber \\  &   \quad +O((\Delta \alpha _s)^2)= T^{(0)}(\alpha _s^{0}) + \lambda \beta , \end{aligned}$$

with

2.21 $$\begin{aligned} \beta = \frac{\partial T^{(0)}\left( \alpha _s\right) }{\partial \alpha _s}\Bigg |_{\alpha _s=\alpha _s^{0}}= \frac{\Delta T^\pm }{\Delta \alpha _s}+O((\Delta \alpha _s)^2), \end{aligned}$$

where

2.22 $$\begin{aligned} \Delta T^\pm = T^{(0)}(\alpha _s^\pm ) - T^{(0)}(\alpha _s^{0}). \end{aligned}$$

Since the dependence of the theory predictions on $\alpha _s$ is slightly non-linear, we approximate the theory covariance matrix used in the fit by averaging over the positive and negative variations:

2.23 $$\begin{aligned} S_{ij} = \beta _{i}\beta _{j}(\Delta \alpha _s)^2 = \frac{1}{2} \left( \Delta T_i^+\Delta T_j^+ +\Delta T_i^-\Delta T_j^- \right) .\nonumber \\ \end{aligned}$$

We have checked that our results are independent of this prescription, provided $\Delta \alpha _s$ is sufficiently small: in practice just a few percent of $\alpha _s,$ so that corrections are of order tenths permille.

Once we have determined *S*,  we simply perform a single PDF determination in which generation of data replicas and fitting using the loss function are both performed using the combined covariance matrix $C+S.$ The additional theory covariance matrix *S* included in this fit allows the PDFs to accommodate the prior uncertainty in $\alpha _s,$ and evaluating the nuisance parameters replica by replica gives us the posterior distribution. Specifically, using Eqs. (2.16) and (2.17), the best-fit value is

2.24 $$\begin{aligned} \bar{\alpha }_s = \alpha _s^{0} +\overline{\lambda }^{(0)} = \alpha _s^{0} + \beta ^T \left( C+S\right) ^{-1} \left( D- T^{(0)} \right) \Delta \alpha _s ,\nonumber \\ \end{aligned}$$

while the associated standard deviation is given by

2.25 $$\begin{aligned} \sigma _\alpha = \left( 1 - \beta ^T \left( C+S\right) ^{-1}\beta +\beta ^T \left( C+S\right) ^{-1} X \left( C+S\right) ^{-1}\beta \right) ^{1/2} \Delta \alpha _s.\nonumber \\ \end{aligned}$$

Independence of the final value of the choice of prior can be achieved by iteration of the whole procedure, with the central value $\alpha _s^{0}$ of the new prior taken as the posterior value $\bar{\alpha }_s$ of the previous fit.

### Settings for $\alpha _s$ extraction

We use both the CRM and TCM for $\alpha _s$ determination, and show that they lead to consistent results. For the CRM, we determine correlated replicas for values of the strong coupling in the range $\alpha _s(m_Z)\in [0.114, 0.125]$ with increments of $\Delta \alpha _s=0.001.$ For the TCM we choose $\Delta \alpha _s=0.002$ for the prior while the central value of the prior is updated iteratively until the prior and posterior agree. Prior independence is also explicitly checked. It is clear that the TCM is generally, and especially with these choices, computationally more efficient than the CRM, since once the theory covariance matrix is computed it requires only a single fit and the evaluation of the formulae Eqs. (2.24, 2.25), as opposed to performing a large number of correlated fits equal to the set of chosen discrete $\alpha _s$ values in the CRM. Moreover, the TCM only requires the determination of PDFs for a value of $\alpha _s$ close to the physical value, while the CRM requires PDF determinations over a wider range of values, some of which are quite far from the physical value. This may be significant, since the hyperoptimization of the fit parameters is performed only for a single value of $\alpha _s$ (0.118 in NNPDF4.0).

## Closure tests

Closure tests [34, 36] as a means to validate PDF sets were first introduced for the NNPDF3.0 determination [33], subsequently used for NNPDF4.0 [15] and recently adopted by other groups [35]. Here we use them, for the first time, to validate an $\alpha _s$ determination: we generate “synthetic” data with a known underlying true value of $\alpha _s$ and the PDFs, and we then show that the value of $\alpha _s$ obtained by running our methodology, blind to this underlying value, agrees with it. This provides us with an extremely stringent test, because in the context of a closure test it is possible to regenerate synthetic data $N_r$ times, corresponding to $N_r$ “runs of the universe”. It is then possible not only to check that the values of $\alpha _s$ obtained in each run of the universe are distributed about the true value according to their nominal uncertainty $\sigma _\alpha ,$ but also that the mean over runs of the universe agrees with the true value with a smaller uncertainty $\sigma _\alpha /\sqrt{N_r}.$ In fact, the closure test has allowed us to detect two possible sources of bias: first, related to the treatment of multiplicative uncertainties, and second, to positivity constraints.

### Closure testing methodology and settings

**Methodology.** Closure tests are performed by generating synthetic data (referred to as L1 data) according to a known underlying law: in our case a known set of PDFs and value of $\alpha _s.$ The true values are referred to as L0 data, and the L1 data are distributed about them according to the full experimental covariance matrix. The NNPDF methodology is then run on these synthetic data: this in particular involves generating Monte Carlo data replicas, referred to as L2 data. The whole procedure is repeated $N_r$ times, corresponding to $N_r$ independent runs of the universe. Hence, $N_r$ sets of L1 data are generated, from each of which we extract a value of $\alpha _s$ using the CRM and the TCM, as discussed in Sect. 2. For reasons of computational cost (see Sect. 2.3) the CRM is run for a smaller number $N_r$ of L1 runs than the TCM.

The accuracy of the results for $\alpha _s$ obtained by each methodology can be tested using the bias-variance ratio or mean normalized bias [34, 36]:

3.1 $$\begin{aligned} {\mathcal {R}}_{\textrm{bv}} = \sqrt{\frac{1}{N_r} \sum _{j=1}^{N_r} \left( {\mathcal {R}}_{\textrm{bv}}^{(j)}\right) ^2}, \end{aligned}$$

with

3.2 $$\begin{aligned} \quad {\mathcal {R}}_{\textrm{bv}}^{(j)}= \frac{\alpha _s^{(j)} - \bar{\alpha }_s}{\sigma _\alpha ^{(j)}}, \end{aligned}$$

where $\bar{\alpha }_s$ is the true underlying value of $\alpha _s,$ and $\bar{\alpha }_s^{(j)}$ and $\sigma _\alpha ^{(j)}$ are respectively the central value and uncertainty on $\alpha _s$ obtained in the *j*-th run of the universe and the sum runs over the $N_r$ runs of the universe. The bias-variance ratio of Ref. [34], and the normalized bias of Ref. [36], when considering several correlated quantities differ in the treatment of correlations, but coincide when considering a single quantity as in our case. The normalized bias ${\mathcal {R}}_{\textrm{bv}}^{(j)}$ should follow a univariate normal distribution, of which the bias-variance ratio is the variance, and should thus equal one for perfectly faithful uncertainties. The uncertainty on ${\mathcal {R}}_{\textrm{bv}}$ can be estimated via the bootstrap method (see e.g. Ref. [36]).

**Settings.** We generate data selecting as true underlying value

3.3 $$\begin{aligned} \bar{\alpha }_s(m_Z) = 0.118 , \end{aligned}$$

and the central value of a (QCD-only) NLO PDF as the true underlying PDF. Since the closure test assumes that the data exactly reproduces the predictions, the particular choice of underlying theory is immaterial and there are no missing higher order contributions and associate uncertainties.

We perform $N_r=25$ determinations of $\alpha _s$ using the CRM, each based on a set of $N_{\text {rep}} = 250$ replicas for each of the 12 values of $\alpha _s$ under consideration (see Sect. 2.3). With the TCM we perform $N_r=100$ determinations, each based on a set of $N_{\text {rep}} = 550$ replicas. These numbers are before the post-fit selection to filter outliers [15].

### Results

**Table 1:** Results of the closure test for the $\alpha _s$ determination performed with the CRM and TCM with different settings. From top to bottom, we show results obtained with a covariance matrix that depends on $\alpha _s,$ with fixed covariance matrix, and without positivity (see text). In each case we show the mean $\langle \alpha _s\rangle$ Eq. (3.4), the uncertainty of the mean, which is by a factor $\sqrt{N_r}$ smaller than the mean uncertainty $\sigma _\alpha$ Eq. (3.5) on the value found in each run, the pull Eq. (3.6) and the bias-variance ratio Eq. (3.1), with uncertainty estimated via the bootstrap method

Method	Settings	$\langle \alpha _s(m_Z)\rangle$	$\langle \sigma _\alpha \rangle /\sqrt{N_r}$	Pull *P*	${\mathcal {R}}_{\textrm{bv}}$
CRM	$C(\alpha _s)$	0.119450	0.000077	19	$3.8 \pm 0.16$
CRM	Fixed *C*	0.118152	0.000070	2.2	$0.97 \pm 0.11$
TCM	Fixed *C*	0.118132	0.000039	3.4	$0.80 \pm 0.06$
CRM	Fixed *C*, no positivity	0.118029	0.000077	0.38	$0.80 \pm 0.09$
TCM	Fixed *C*, no positivity	0.117984	0.000041	0.39	$0.71 \pm 0.05$

**Methodological choices.** We have considered a large number of possible methodological choices and variations, both concerning the NNPDF methodology in general, and the $\alpha _s$ determination in particular, in order to assess whether any of them would affect the faithfulness of the $\alpha _s$ value. Specifically, with the CRM in the determination of the best-fit $\alpha _s,$ Eq. (2.4), we interpolated the available discrete values of $E^{(k)}(\overline{\theta }^{(k)}(\alpha _s),\alpha _s)$ with polynomials of increasingly higher order; we used $\ln \alpha _s$ instead of $\alpha _s$ as a variable; we checked the effect of following the multi-batch procedure of Ref. [8] in which each data replica is fitted several times and the best fit is selected vs. a single-batch. None of these variations had any significant effect [44]. In the TCM, we significantly increased the width of the prior, with no visible effect. For both CRM and TCM we also generated L2 data using either the experimental covariance matrix or the $t_0$ covariance matrix (see Ref. [45], specifically Table 9, for a discussion of the difference between the two); Again, this variation did not have any significant effect.

However, we did find two methodological choices that do have an impact on the determination of $\alpha _s,$ namely the treatment of multiplicative uncertainties in the experimental covariance matrix and the treatment of positivity. We discuss each of them in turn.

**Multiplicative Uncertainties** Both the experimental and theoretical covariance matrix $C^{\textrm{exp}}_{t_0}$ and $C^{\textrm{th}}$ Eq. (2.2) depend on the value of $\alpha _s.$ Indeed, the $t_0$ experimental covariance matrix $C^{\textrm{exp}}_{t_0}$ [41] is computed using the theory predictions from a previous fit to determine multiplicative uncertainties, and the theory covariance matrix $C^{\textrm{th}}$ is found performing scale variations, whose size is manifestly dependent on the value of $\alpha _s.$

It must consequently be decided whether, when varying the value of $\alpha _s$ in the theory prediction used to determine its best-fit, the value of $\alpha _s$ in the computation of the covariance matrix should also be varied, or not. In the closure test, of course, as there is no MHOU, only the effect of this choice for $C^{\textrm{exp}}_{t_0}$ is relevant. The test is most easily performed in the CRM, where the theory predictions are computed for a fixed set of value of $\alpha _s,$ and a loss $E^{(k)}(\overline{\theta }^{(k)}(\alpha _s),\alpha _s)$ Eq. (2.4) is then determined for each value. The question is then whether the same covariance matrix should be used when computing the loss for each value of $\alpha _s,$ or whether the covariance matrix should be re-determined for each value of $\alpha _s$ along with the theory prediction.

The value of $\alpha _s$ obtained when varying the covariance matrix as a function of $\alpha _s$ is shown in the first row of Table 1. We display there the weighted mean over the $N_r$ runs

3.4 $$\begin{aligned} \langle \alpha _s(m_Z) \rangle =\frac{\sum _{j=1}^{N_r} \frac{\alpha ^{(j)}_s(m_Z)}{\left( \sigma ^{(j)}_\alpha \right) ^2}}{\sum _{j=1}^{N_r}\frac{1}{\left( \sigma ^{(j)}_\alpha \right) ^2}}, \end{aligned}$$

where the weighted uncertainty is

3.5 $$\begin{aligned} \langle \sigma _\alpha \rangle = \frac{1}{\sqrt{\sum _{j=1}^{N_r} \frac{1}{\left( \sigma ^{(j)}_\alpha \right) ^2}}}. \end{aligned}$$

In the same table we also show the uncertainty of the mean, given by $\langle \sigma _\alpha \rangle /\sqrt{N_r},$ the pull

3.6 $$\begin{aligned} P=\frac{\frac{1}{N_r}\sum _{j=1}^{N_r}\left( \alpha _s^{(j)} - \bar{\alpha }_s\right) }{\langle \sigma _\alpha \rangle /N_r}, \end{aligned}$$

and the bias-variance ratio. It is clear that the closure test fails: the bias-variance ratio shows that the deviation of results from truth is on average four times bigger than the nominal uncertainty. Note that the pull is correspondingly $P\approx \sqrt{N_r}{\mathcal {R}}_{\textrm{bv}}\approx 20.$

The value of $\alpha _s$ extracted when keeping the covariance matrix fixed, shown in the second row of Table 1, is instead free of this problem. The bias-variance ratio is now somewhat smaller than one, indicating that the mean-square deviation of $\alpha _s$ is consistent with its stated uncertainty, with, in fact, a slight uncertainty overestimation. This agrees with the result found in Ref. [36] for PDFs. The value of $\alpha _s$ found using the TCM, where the covariance matrix is kept fixed by construction since only the dependence on $\alpha _s$ through the theory predictions is included in Eq. (2.20), is given in the third row of the table, and it is also in agreement, with a bias-variance ratio less than one. We have also checked that the same consistent result is reproduced if all uncertainties are treated as additive. Indeed, in this case the $t_0$ matrix is not used at all, so the covariance matrix becomes independent of $\alpha _s.$

This somewhat counter-intuitive result can be explained by noting that recomputing the covariance matrix as a function of $\alpha _s$ introduces a dependence of the experimental correlated systematics on $\alpha _s.$ Since many hadronic cross-sections increase as $\alpha _s$ increases, this then tends to make multiplicative uncertainties larger for larger $\alpha _s,$ and thus the loss smaller, thereby leading to an upward bias in the best-fit value. We conclude that a consistent determination of $\alpha _s$ requires keeping the covariance matrix fixed as $\alpha _s$ is varied, a result that would have been difficult to establish without the closure test.

**Positivity** Closer inspection of Table 1 reveals that while the value of $\alpha _s$ determined with fixed covariance matrix deviates on average from the true value by an amount which is consistent with its nominal uncertainty, it nevertheless displays a pull *P* well above one. We have checked that this pull remains approximately constant when increasing the number $N_r$ of runs: the deviation of the mean from the truth decreases but so does its uncertainty. This means that whereas in each run the deviation of the best-fit $\alpha _s$ from truth is consistent with its uncertainty (because $R_{bv}\sim 1$), the distribution of best-fits about the true value is asymmetric, so on average biased. In a closure test this bias can be reduced by increasing the number $N_r$ of runs, but a real-world determination of course will consist of a single run, hence it is important to understand the origin of the bias.

**Fig. 1:**
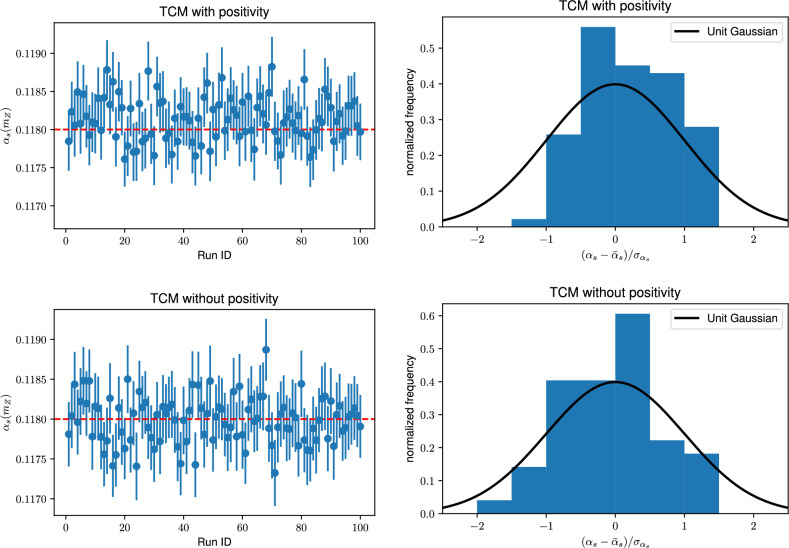
The best-fit values of $\alpha _{s}^{(j)}$ and the associated one standard deviation uncertainties $\sigma ^{(j)}_\alpha$ obtained using the TCM in the $N_r=100$ individual runs of the closure tests (left), and the corresponding distribution of normalized bias ${\mathcal {R}}_{\textrm{bv}}^{(j)}$ Eq. (3.2) (right). For reference, a univariate zero-mean Gaussian is also displayed in the right panels. Results obtained both when imposing positivity (top) and when not imposing it (bottom) are shown

We have traced this bias to the positivity constraints imposed in the PDF fit, see Sect. 3.1.3 of Ref. [15] for a detailed discussion. Results found both with the TCM and CRM after removing these constraints are shown in Table 1. It is clear that while the bias-variance ratio is unchanged, the pull is now below one, showing that the bias has disappeared. Also, in Fig. 1 we compare the results obtained with and without positivity for the individual $N_r$ determinations of $\alpha _s(m_Z)$ using the TCM. We show both a comparison of the result with its central value $\alpha _s^{(j)}$ and uncertainty $\sigma _\alpha ^{(j)}$ to the underlying truth $\bar{\alpha }_s=0.118,$ and the histogram of normalized bias ${\mathcal {R}}_{\textrm{bv}}^{(j)},$ Eq. (3.1), superposed to a univariate Gaussian, which is its expected distribution.

It is clear from the figure that the distribution of $\alpha _s^{(j)}$ values is to the same good approximation Gaussian with or without positivity, in agreement with the fact that the bias-variance ratio with and without positivity remains the same: the distribution of results about the mean is in each case compatible with its uncertainty and symmetric. However, the distribution of results about the true value without positivity is also symmetric, while with positivity it is biased, as it is clear from Fig. 1 (left) where it is clear that with positivity the number of values of $\alpha _s^{(j)}$ above the horizontal line is larger than the number of values below. We conclude that positivity results in a bias that produces an offset of the center of the distribution of $\alpha _s^{(j)}$ values with respect to the true value.

The impact of positivity can be understood by noting that in the vicinity of kinematic boundaries the data uncertainty is necessarily non-Gaussian, because a Gaussian always has an infinite tail which extends in the region of negative cross-sections. However, experimental data uncertainties are assumed to be Gaussian and treated as such in the data replica generation, which may generate negative data replicas. This may then lead to an inconsistency between the distribution of optimized PDF replicas, which are constrained to lead to positive predictions, and that of the underlying data.

Tracing which datasets lead to the effect is however difficult, since all data are correlated through their PDF dependence, PDFs are in turn correlated by the momentum sum rule, and there might be an interplay between data, and theoretical positivity constraints that are also imposed in the fit [15] in order to ensure that physical observables remain positive even outside the data region.

**Fig. 2:**
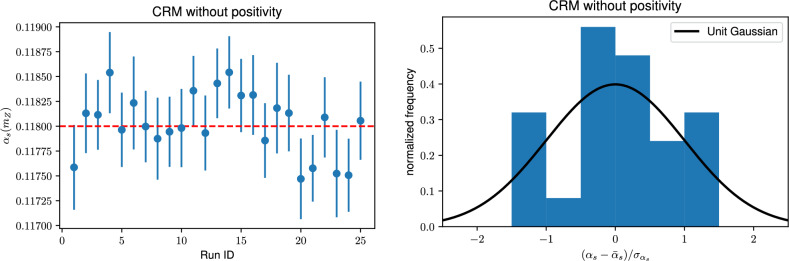
Same as Fig. 1 (bottom) but now for results obtained in the $N_r=25$ runs of the CRM

**Closure Results.** The two determinations of $\alpha _s$ shown in Table 1 after removing positivity constraints (bottom two entries) satisfy the closure test. Note that the central values of the two determinations are not exactly the same since the CRM result is determined from $N_r=25$ runs and the TCM result from $N_r=100$ runs. However, the average uncertainty $\langle \sigma _\alpha \rangle$ Eq. (3.5) agrees: the two determinations have the same pull, well below one. The agreement of results found with the two methods is also demonstrated by repeating the TCM plot of Fig. 1, but now using the CRM, see Fig. 2

Note also that the uncertainty on the CRM prediction is determined for each run from the variance of the set of $N_{\textrm{rep}}$ $\alpha _s^{(k)}$ values Eq. (2.4), while the uncertainty on the TCM prediction is found using the analytic formula Eq. (2.25). We have checked that the result for the TCM uncertainty found using the analytic formula agrees with that computed from the standard deviation over the replica sample, and that the 68% CL over replicas differs only at the sub-permille level. This indicates that the distribution of $\alpha _s$ found using the TCM is Gaussian to very good approximation, as expected.

Finally, we test for independence of the prior of results obtained using the TCM. This is especially important in view of the fact that in the previous tests we always used a prior centered at the true value. To this purpose, we have determined the posterior value of $\alpha _s$ by taking as a prior $\alpha _s(m_Z)=0.117$ or $\alpha _s(m_Z)=0.119.$ We respectively find $\alpha _s(m_Z)=0.11801$ and $\alpha _s(m_Z)=0.11811$ as posterior values. This proves that the method converges rapidly: the subsequent iteration would then essentially coincide with our previous TCM determination, thereby confirming prior independence. Note that in all cases, as discussed in Sect. 2.2 the width of the prior is $\Delta \alpha _s=0.002,$ hence it is much wider than the positivity bias, i.e. the shift of the best-fit central value due to positivity.

We conclude that the closure test is successful: the CRM and the TCM lead to results in agreement with each other, unbiased and with faithful uncertainties. We further conclude that unbiased results are obtained with a covariance matrix that does not vary with $\alpha _s,$ and in the absence of positivity constraints. In the presence of positivity constraints, results are still Gaussianly distributed, but converge to biased result, offset by a positive amount with respect to the true value.

## The strong coupling at ${\textrm{aN}}^3$LO accuracy

We now present the main result of this work, namely the extraction of $\alpha _s(m_Z)$ up to ${\textrm{aN}}^3$LO QCD and NLO QED accuracy. First we present our baseline determination, discussing its methodological aspects and perturbative stability and providing our assessment of its total uncertainty. We then check that our error estimate is robust, by verifying that our result is stable upon various possible methodological variations. We finally compare our result to previous determinations both by us and by other groups.

**Table 2:** Baseline results for $\alpha _s(m_Z)$ obtained at different perturbative orders, using the CRM and TCM respectively discussed in Sects. 2.1 and 2.2. The final column gives our best value at each perturbative order, obtained using the TCM result. The asymmetric uncertainty is obtained by accounting for the systematic uncertainty associated to the positivity bias (see text)

Perturbative order	TCM	CRM	Best value
${\textrm{NNLO}}_{\textrm{QCD}}$	$0.1198 \pm 0.0008$	$0.1199 \pm 0.0006$	$0.1198^{+0.0007}_{-0.0010}$
${\textrm{NNLO}}_{\textrm{QCD}}\otimes$ ${\textrm{NLO}}_{\textrm{QED}}$	$0.1203 \pm 0.0007$	$0.1201 \pm 0.0006$	$0.1203^{+0.0007}_{-0.0010}$
${\textrm{aN}}^3$ ${\textrm{LO}}_{\textrm{QCD}}$	$0.1192 \pm 0.0007$	$0.1191 \pm 0.0008$	$0.1192^{+0.0007}_{-0.0013}$
${\textrm{aN}}^3$ ${\textrm{LO}}_{\textrm{QCD}}\otimes$ ${\textrm{NLO}}_{\textrm{QED}}$	$0.1194 \pm 0.0007$	$0.1194 \pm 0.0007$	$0.1194^{+0.0007}_{-0.0014}$

### Baseline results

Our baseline results are obtained using the same NNPDF4.0 dataset, methodology and theory predictions as in Refs. [21, 23, 26, 46]. Theory predictions use the pipeline described in Ref. [47] which is built upon the EKO [48, 49] evolution code and the PineAPPL fast grid interface [50, 51]. These results always include MHOUs on the theory prediction as discussed in Ref. [26], and, in each case, with NNLO or ${\textrm{aN}}^3$LO QCD theory, and with or without QED corrections. QED corrections are included according to Ref. [23], and ${\textrm{aN}}^3$LO QCD corrections following Ref. [21], updated with the most recent implementation of heavy quark matching of Ref. [52]. The value of $\alpha _s$ is extracted using the CRM and the TCM, respectively discussed in Sects. 2.1 and 2.2, with the settings discussed in Sect. 2.3, and the same number of replicas used in each closure test run (see Sect. 3.1), namely for the CRM $N_{\text {rep}} = 250$ replicas for each of the 12 values of $\alpha _s$ under consideration, and $N_{\text {rep}} = 550$ replicas for the TCM. As with the closure test, these are the numbers of replicas before the post-fit selection used to filter outliers [15]. The number of replicas are chosen such that the finite-size uncertainty as estimated through bootstrapping is less than one permille on the central value of the extracted $\alpha _s.$ Uncertainties are determined both as one-sigma and 68% CL intervals from the replica sample, and for the TCM also using the analytic formula Eq. (2.25), with results always agreeing to within the number of decimal figures shown in the table (i.e. at the permille level). Because we include both experimental uncertainties and MHOUs, and we simultaneously determine $\alpha _s$ and the PDFs, the resultant uncertainty includes methodological, experimental and theoretical (nuclear and MHO) uncertainties, though not the systematic uncertainty related to the positivity bias, detected in the closure test of Sect. 3.2 and further discussed below. Results are collected in Table 2.

**Methodology variations.** Inspection of Table 2 shows that the TCM and CRM results are always in agreement, with differences in central values and uncertainties at the permille level. We have also repeated all determinations using the deprecated method discussed at the end of Sect. 2.1 and based on Eqs. (2.5)–(2.7), neglecting the correlation between $\alpha _s$ and the PDF as in Refs. [11, 43]. We have verified that this leads to the same central values, also at the permille level, but to an underestimate of the uncertainty by up to 40%. We have also recomputed all values while excluding the MHOUs [26], as is done at NNLO by all other groups. This leads to an underestimate of the uncertainty which at NNLO can be up to a factor of two, with an associated shift in the central value of about one sigma: upwards at NNLO and downwards at ${\textrm{aN}}^3$LO. Specifically we find that the uncertainty on the pure QCD result increases from $\pm 0.0004$ to $\pm 0.0008$ at NNLO and from $\pm 0.0006$ to $\pm 0.0007$ at ${\textrm{aN}}^3$LO, suggesting that the MHOU is about $\pm 0.0007$ at NNLO and $\pm 0.0004$ at ${\textrm{aN}}^3$LO.

**Table 3:** Same as Table 2 but removing the positivity constraint on physical observables

Perturbative order	TCM	CRM
${\textrm{NNLO}}_{\textrm{QCD}}$	$0.1195 \pm 0.0008$	$0.1203 \pm 0.0008$
${\textrm{NNLO}}_{\textrm{QCD}}\otimes$ ${\textrm{NLO}}_{\textrm{QED}}$	$0.1200 \pm 0.0008$	$0.1204 \pm 0.0010$
${\textrm{aN}}^3$ ${\textrm{LO}}_{\textrm{QCD}}$	$0.1186 \pm 0.0009$	$0.1191 \pm 0.0009$
${\textrm{aN}}^3$ ${\textrm{LO}}_{\textrm{QCD}}\otimes$ ${\textrm{NLO}}_{\textrm{QED}}$	$0.1187 \pm 0.0009$	$0.1194 \pm 0.0008$

**Perturbative stability and QED corrections.** The results shown in Table 2 show perturbative stability: the value of $\alpha _s$ decreases as the perturbative order increases, as previously observed when going from NLO to NNLO [8], but the results at two subsequent orders agree at the one sigma level, as they ought to given that MHOUs are included. Indeed, if MHOUs are not included the NNLO and ${\textrm{aN}}^3$LO values (CRM, pure QCD) become respectively $\alpha _s(m_Z)=0.1205\pm 0.0004$ and $\alpha _s(m_Z)=0.1187\pm 0.0006,$ and hence disagree at the four–five sigma level. Despite the fact that MHOUs contribute substantially to the overall uncertainty, the total uncertainty does not decrease when going from NNLO to ${\textrm{aN}}^3$LO. This is unsurprising as at ${\textrm{aN}}^3$LO corrections are only included for perturbative evolution [48, 49] and deep-inelastic coefficient functions [53, 54] while for all hadronic processes, which carry substantial weight in determining $\alpha _s,$ partonic cross-sections are still computed at NNLO with corresponding MHOUs.

The inclusion of QED corrections has the effect of increasing the value of $\alpha _s$ by a small but non-negligible amount. This can most likely be understood as a consequence of the fact that the photon PDF subtracts momentum from the gluon, and the ensuing slight suppression of the gluon is compensated by a slightly larger value of $\alpha _s.$ It is important to observe that, to the best of our knowledge, the effect of QED corrections is not included in any other simultaneous determination of $\alpha _s$ and PDFs, and, moreover, the associated uncertainty is clearly not included in QCD scale variation and therefore routinely neglected.

**Impact of positivity.** We have found from the closure test analysis of Sect. 3 that imposing positivity constraints leads to a bias in the extracted value of $\alpha _s(m_Z).$ Therefore, we repeated the determinations shown in Table 2, but now removing this positivity constraint. In this case, the CRM result corresponding to the outer $\alpha _s$ values become somewhat unstable: specifically we have verified that the 68% CL and one-sigma uncertainties are significantly different, and we have traced this to the presence of outliers in the replica distribution. We have consequently run two batches according to the method of Ref. [8]: for each data replica two fits are performed, and that leading to the best loss is chosen. However, the non-Gaussianity persists also in this case.

Results for the extraction of $\alpha _s$ when the positivity constraints are not imposed are shown in Table 3, with the same theory settings as in Table 2. It is clear that just like in the closure test, removing positivity constraints leads to a downward shift of the $\alpha _s$ value. As discussed in Sect. 3.2 the effect of positivity may be understood as a consequence of the non-Gaussian nature of uncertainties in the vicinity of kinematic boundaries. However, experimental uncertainties are delivered as Gaussian and consequently we cannot easily correct for this. Moreover, because the TCM and the CRM in the absence of positivity no longer agree, it is not easy to estimate reliably the size of the bias. We have therefore conservatively taken the difference between the TCM result with and without positivity as an extra source of uncertainty. We use the TCM result since, in most cases, the shift due to positivity is larger than the corresponding shift of the CRM result. This source of uncertainty is considered to reflect a non-Gaussian bias, and thus added linearly. Also, since relaxing positivity in the TCM always produces a downward shift, this contribution is added only to the lower uncertainty, resulting in an asymmetric overall uncertainty.

**Fig. 3:**
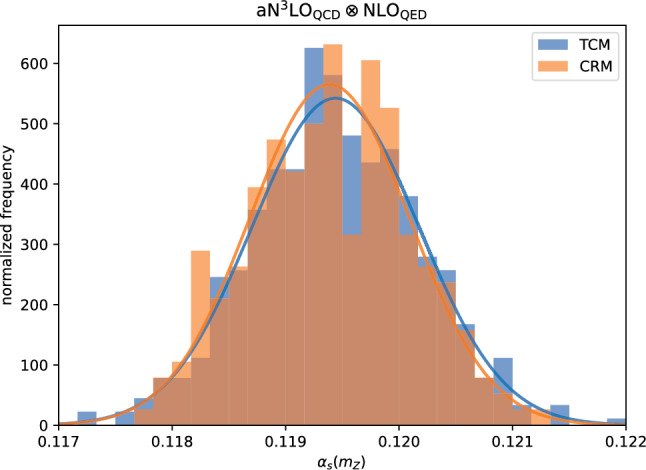
Histogram of the values of the $N_{\textrm{rep}}$ best-fit values $\alpha _s^{(k)}$ obtained with the TCM and CRM when applied to the experimental data entering the NNPDF4.0 global fit. In both cases, results shown correspond to the fits carried out at ${\textrm{aN}}^3$ ${\textrm{LO}}_{\textrm{QCD}}\otimes$ ${\textrm{NLO}}_{\textrm{QED}}$ accuracy and accounting for the positivity of physical observables, see the bottom row of Table 2 for the corresponding central values and 68% CL uncertainties on $\alpha _s(m_Z).$ The curves are Gaussian fits to the two distributions

**Final results.** The histogram of the ensemble of $\alpha _s$ replica values obtained using the CRM and TCM in the fits with ${\textrm{aN}}^3$ ${\textrm{LO}}_{\textrm{QCD}}\otimes$ ${\textrm{NLO}}_{\textrm{QED}}$ accuracy and accounting for the positivity constraints is displayed in Fig. 3. It is clear that the distributions are both Gaussian and in excellent agreement. We take as our best value for $\alpha _s$ and its uncertainty that obtained with the TCM, which is based on a larger number of replicas. The final uncertainty on this value is determined by adding linearly to the lower uncertainty the difference between the TCM results with and without positivity at the corresponding perturbative order. The final results are collected in the last column of Table 2.

**Table 4:** Results for the determination of $\alpha _s$ obtained with two variations of methodological settings: using the expanded instead of the exact solution of evolution equations, and using the experimental covariance matrix instead of the $t_0$ covariance matrix for the data replica generation (see text). For the former, we only compare results of the fits with ${\textrm{NNLO}}_{\textrm{QCD}}$ and ${\textrm{aN}}^3$ ${\textrm{LO}}_{\textrm{QCD}},$ given that the inclusion of QED corrections requires the use of the exact solution [23]

Perturbative order	Theory setting	TCM	CRM
${\textrm{NNLO}}_{\textrm{QCD}}$	Expanded solution	$0.1195 \pm 0.0007$	$0.1196 \pm 0.0006$
${\textrm{aN}}^3$ ${\textrm{LO}}_{\textrm{QCD}}$	Expanded solution	$0.1192 \pm 0.0007$	$0.1194 \pm 0.0007$
${\textrm{NNLO}}_{\textrm{QCD}}$	Exp. covmat replicas	$0.1199 \pm 0.0007$	$0.1199 \pm 0.0006$
${\textrm{NNLO}}_{\textrm{QCD}}\otimes$ ${\textrm{NLO}}_{\textrm{QED}}$	Exp. covmat replicas	$0.1202 \pm 0.0006$	$0.1201 \pm 0.0006$
${\textrm{aN}}^3$ ${\textrm{LO}}_{\textrm{QCD}}$	Exp. covmat replicas	$0.1192 \pm 0.0007$	$0.1191 \pm 0.0007$
${\textrm{aN}}^3$ ${\textrm{LO}}_{\textrm{QCD}}\otimes$ ${\textrm{NLO}}_{\textrm{QED}}$	Exp. covmat replicas	$0.1194 \pm 0.0007$	$0.1195 \pm 0.0007$

**Fig. 4:**
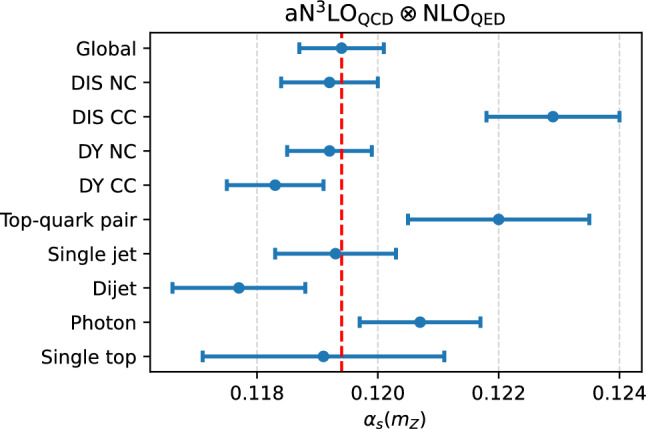
The values of $\alpha _s(m_Z)$ extracted at ${\textrm{aN}}^3$ ${\textrm{LO}}_{\textrm{QCD}}\otimes$ ${\textrm{NLO}}_{\textrm{QED}}$ accuracy from the TCM applied to the partial $\chi ^2$ evaluated for separate groups of processes. In all cases, uncertainties shown correspond to 68% CL intervals. The dashed vertical line corresponds to the best-fit value obtained from the global dataset

### Methodological stability

**Solution of evolution equations.** In our default determination of $\alpha _s,$ the QCD and QCD$\otimes$QED evolution equations are solved in the same way. This requires using an exact solution, rather than the expanded solution which was previously used by us for pure QCD evolution in Refs. [15, 21], because construction of the expanded solution for QCD$\otimes$QED evolution is problematic: see Ref. [23] for a detailed discussion. Differences between the two methods are subleading in the QCD expansion. In Table 4 we show the results obtained by switching to the expanded solution in the pure QCD determinations at NNLO and ${\textrm{aN}}^3$LO. Results are shown to change by less than half a sigma at NNLO, and to be essentially unchanged at ${\textrm{aN}}^3$LO. The decrease of the NNLO result when using truncated evolution reduces the difference between NNLO and ${\textrm{aN}}^3$LO by a factor of two.

**Table 5:** Comparison of the results of the NNPDF4.0 determination of $\alpha _s(m_Z)$ presented in this work (first row) with other recent determinations of the strong coupling. See Fig. 5 for a graphical representation of the results

Determination	Perturbative accuracy	Dataset	$\alpha _s(m_Z)$	Refs.
NNPDF4.0	${\textrm{aN}}^3$ ${\textrm{LO}}_{\textrm{QCD}}\otimes$ ${\textrm{NLO}}_{\textrm{QED}}$	Global	$0.1194^{+0.0007}_{-0.0014}$	This work
NNPDF3.1	${\textrm{NNLO}}_{\textrm{QCD}}$	Global	$0.1185 \pm 0.0012$	[8]
MSHT20	${\textrm{NNLO}}_{\textrm{QCD}}$	Global	$0.1171 \pm 0.0014$	[55]
MSHT20	${\textrm{aN}}^3$ ${\textrm{LO}}_{\textrm{QCD}}$	Global	$0.1170 \pm 0.0016$	[10]
ABMPtt	${\textrm{NNLO}}_{\textrm{QCD}}$	Global (no jets)	$0.1150\pm 0.0009$	[56]
ATLAS $p_T^Z$ 8 TeV	${\textrm{N}}^{3}{\textrm{LO}}\otimes {\textrm{N}}^4\textrm{LLa}_{\textrm{QCD}}$	$d\sigma (Z\rightarrow \ell ^+\ell ^-)/dp_T^Z$	$0.1183 \pm 0.0009$	[57]
CMS jets 13 TeV	${\textrm{NNLO}}_{\textrm{QCD}}$	$d^2\sigma /dp_T^jdy_j$	$0.1166 \pm 0.0017$	[58]
ALPHA 25 (lattice QCD)	–	–	$0.11873 \pm 0.00056$	[59]
PDG 2024	–	Average	$0.1180 \pm 0.0009$	[3]
PDG 2024 (no lattice QCD)	–	Average excl. lattice	$0.1175 \pm 0.0010$	[3]

**Fig. 5:**
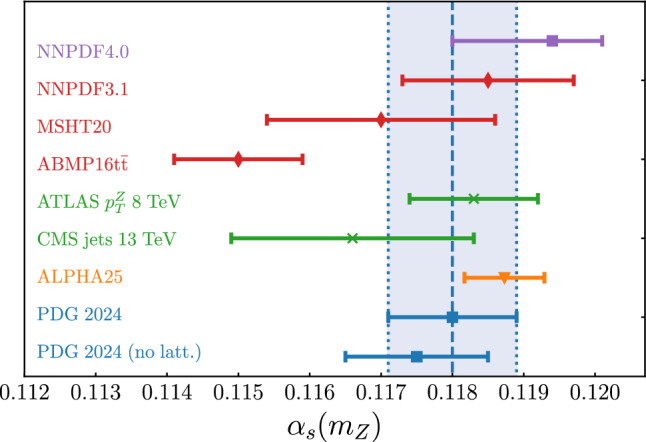
Graphical representation of the results of Table 5. For MSHT20, we show the ${\textrm{aN}}^3$LO QCD result. The filled vertical band corresponds to the 2024 PDG average of $\alpha _s(m_Z)=0.1180 \pm 0.0009$

**Data replica generation.** When generating Monte Carlo data replicas (see Sect. 2.1) one may choose to use the experimental covariance matrix, or the $t_0$ covariance matrix, as discussed in Sect. 3.1, where it was mentioned that independence of results on this choice was explicitly checked. In Table 4 we also show results obtained by performing this methodological variation. Comparing to Table 2 shows that indeed results are all but unaffected by this choice.

**Value of the top quark mass.** Our global dataset includes top production data, for which the theoretical predictions are sensitive to the value of the top quark mass. In our default NNPDF4.0 determination [15] we adopt the value $m_t=172.5$ GeV for the pole top mass. We have repeated our NNLO pure QCD determination with $m_t=175.0$ GeV and $m_t=170.0$ GeV. This corresponds to a variation of almost four times the PDG pole mass uncertainty of $\Delta m_t=0.7$ GeV [3]. Within this wide range we find that the value of $\alpha _s$ changes by $\Delta \alpha =0.0004$ at NNLO and $\Delta \alpha =0.0001$ at ${\textrm{aN}}^3$LO, increasing with increasing top mass. We conclude that our result is essentially independent of the value of the top quark mass. This is likely a consequence of the fact that the top pair production data constitute a relatively small subset of our global dataset, in particular when compared to other gluon-sensitive measurements such as single-inclusive jets and dijets.

### Comparison to other determinations

**Dataset dependence.** Figure 4 displays the values of $\alpha _s(m_Z)$ extracted at ${\textrm{aN}}^3$ ${\textrm{LO}}_{\textrm{QCD}}\otimes$ ${\textrm{NLO}}_{\textrm{QED}}$ accuracy using the TCM applied to the partial $\chi ^2$ evaluated for separate (exclusive) groups of processes. In all cases, uncertainties shown correspond to 68% CL intervals. The values shown give an indication of the $\alpha _s$ preferred by individual processes. However, they cannot be understood as the best-fit values associate to that process [6], and in particular the global $\alpha _s$ value does not correspond to their weighted mean. This is not only because these values neglect correlations between different processes, but also because for each process there exist in general values of $\alpha _s$ that give a better fit to it while giving the same quality fit to the rest of the dataset [6, 8]. This said, the qualitative indication coming from Fig. 4 is that charged-current deep–inelastic structure functions, direct photon production, and top quark pair production data prefer a larger value of $\alpha _s(m_Z),$ while Drell–Yan charged current and dijet cross-sections instead prefer a lower one.

We have furthermore verified that using the NNPDF3.1-like dataset (as defined in Ref. [15]) and the same theory settings as in Ref. [8], namely pure QCD NNLO theory without MHOUs and with expanded solution of the evolution equation, the value of $\alpha _s$ extracted with the TCM is $\alpha _s(m_Z)=0.1189\pm 0.0005.$ This is to be compared to the value $\alpha _s(m_Z)=0.1185\pm 0.0005$ obtained with the CRM in that reference. Using instead the exact solution and including MHOUs the NNLO value from the NNPDF3.1-like dataset is $\alpha _s(m_Z)=0.1188\pm 0.0006.$ This implies that a substantial part of the difference between the value of $\alpha _s$ of Ref. [8], and the rather higher NNLO value of Table 2 is due to the much larger weight of LHC data in the NNPDF4.0 dataset, and not to any methodological differences, and in particular not at all to differences between the NNPDF3.1 and NNPDF4.0 methodology. The determination of $\alpha _s$ from Ref. [8] was assigned an extra MHOU uncertainty of $\pm 0.0011,$ estimated as half the shift between the NLO and NNLO $\alpha _s$ values, as the formalism of Refs. [29, 30] for the inclusion of MHOUs was not yet available at the time. Interestingly, the MHOU on the NNLO result determined here, $\pm 0.0007,$ see Sect. 4.1, is smaller by almost a factor of two.

**Other **$\alpha _s$** determinations** Table 5 displays the comparison of the results of the NNPDF4.0 extraction of $\alpha _s(m_Z)$ presented in this work, based on ${\textrm{aN}}^3$ ${\textrm{LO}}_{\textrm{QCD}}\otimes$ ${\textrm{NLO}}_{\textrm{QED}}$ theory calculations and accounting for MHOUs, with other recent determinations of the strong coupling jointly with PDFs. Specifically, we compare with the MSHT20 NNLO and ${\textrm{aN}}^3$LO determinations, the ABMPtt updated analysis including differential top quark data, as well as with our previous NNLO determination based on NNPDF3.1. We also include the two single most precise determinations performed by ATLAS and CMS and based on the $p_T$ distributions of *Z* bosons at 8 TeV and on the double-differential single-inclusive jet cross-sections at 13 TeV respectively, though only in the latter PDFs are determined simultaneously with the strong coupling.^1^

We finally display the recent lattice result [59] from the ALPHA collaboration, which is the single most precise determination, and the latest published PDG averages, both global and not including lattice QCD input. See Fig. 5 for the corresponding graphical representation of the results, where for MSHT20 we only display the ${\textrm{aN}}^3$LO QCD result.

All results shown in Table 5 and Fig. 5 overlap within uncertainties among themselves, except the ABMPtt value. Note, however, that the latter presents a simultaneous determination of $\alpha _s$ and the $\overline{\textrm{MS}}$ top mass; if the PDG value of the top mass is used, then a higher value of $\alpha _s$ consistent with the PDF average is obtained [56].

## Summary and outlook

We have presented an extraction of $\alpha _s(m_Z)$ with high precision and accuracy: the width of the (asymmetric) uncertainty band in our determination is the same as that of the PDG combination that excludes lattice data. Our determination of $\alpha _s$ has several unique features, all of which are implemented to the best of our knowledge for the first time in a simultaneous determination of $\alpha _s$ and PDFs:

- The extraction is performed using both frequentist Monte Carlo resampling, and Bayesian inference.
- Its methodology is validated by a closure test.
- Uncertainties include the MHOUs on the processes used for PDF determination both at NNLO and ${\textrm{aN}}^3$LO.
- Effects of mixed QCD$\otimes$QED evolution and the photon PDF are accounted for.

All of these are important for the reliability of the results. Specifically, without the closure test analysis it would have been impossible to detect the bias due to imposing positivity constraints. Without MHOUs the uncertainty on $\alpha _s$ would have been underestimated by up to a factor two. The inclusion of QED corrections affects the central value of $\alpha _s$ at the level of a few permille. We have no reason to believe that these effects would not have a comparable impact if they were studied or included in other simultaneous determinations of PDFs and $\alpha _s.$

It will be interesting in the future to use the methods deployed in this work to carry out joint determinations of PDFs with other physical parameters in addition to $\alpha _s(m_Z),$ such as the top quark mass, and to validate them with closure tests. Also, with the availability of more data it might be interesting to carry out $\alpha _s(Q)$ extractions in separate bins of *Q*,  in order to constrain new physics scenarios which may distort the scale dependence of $\alpha _s(Q)$ in comparison to the standard model prediction [61].

The NNPDF4.0 PDF sets used for this work are available, in the LHAPDF format [37], through the NNPDF website:

https://nnpdf.mi.infn.it/nnpdf4-0-alphas/

Specifically, we release NNLO and ${\textrm{aN}}^3$LO QCD sets, without and with QED corrections, for all values of $\alpha _s(m_Z)$ used for the present determination. All sets are composed of $N_{\textrm{rep}}=200$ replicas. In all cases MHOUs are included, and multiplicative correlated uncertainties are determined using a fixed $t_0$ matrix corresponding to the PDF set at the best $\alpha _s$ which is indicated in the set name, for the reason explained in Sect. 3.1. The replicas are correlated, meaning that replicas with the same index corresponding to different values of $\alpha _s$ are all fitted to the same underlying data replica, see Sect. 2.1.

They are denoted as follows:

- NNLO QCD (+ QED effects)

**Table:** 

NNPDF40_nnlo_as_01140_mhou_t0120	(NNPDF40_nnlo_as_01140_qed_mhou_t0120)
NNPDF40_nnlo_as_01150_mhou_t0120	(NNPDF40_nnlo_as_01150_qed_mhou_t0120)
NNPDF40_nnlo_as_01160_mhou_t0120	(NNPDF40_nnlo_as_01160_qed_mhou_t0120)
NNPDF40_nnlo_as_01170_mhou_t0120	(NNPDF40_nnlo_as_01170_qed_mhou_t0120)
NNPDF40_nnlo_as_01180_mhou_t0120	(NNPDF40_nnlo_as_01180_qed_mhou_t0120)
NNPDF40_nnlo_as_01190_mhou_t0120	(NNPDF40_nnlo_as_01190_qed_mhou_t0120)
NNPDF40_nnlo_as_01200_mhou_t0120	(NNPDF40_nnlo_as_01200_qed_mhou_t0120)
NNPDF40_nnlo_as_01210_mhou_t0120	(NNPDF40_nnlo_as_01210_qed_mhou_t0120)
NNPDF40_nnlo_as_01220_mhou_t0120	(NNPDF40_nnlo_as_01220_qed_mhou_t0120)
NNPDF40_nnlo_as_01230_mhou_t0120	(NNPDF40_nnlo_as_01230_qed_mhou_t0120)
NNPDF40_nnlo_as_01240_mhou_t0120	(NNPDF40_nnlo_as_01240_qed_mhou_t0120)
NNPDF40_nnlo_as_01250_mhou_t0120	(NNPDF40_nnlo_as_01250_qed_mhou_t0120)
- ${\textrm{aN}}^3$LO QCD (+ QED effects)

**Table:** 

NNPDF40_an3lo_as_01140_mhou_t0119	(NNPDF40_an3lo_as_01140_qed_mhou_t0119)
NNPDF40_an3lo_as_01150_mhou_t0119	(NNPDF40_an3lo_as_01150_qed_mhou_t0119)
NNPDF40_an3lo_as_01160_mhou_t0119	(NNPDF40_an3lo_as_01160_qed_mhou_t0119)
NNPDF40_an3lo_as_01170_mhou_t0119	(NNPDF40_an3lo_as_01170_qed_mhou_t0119)
NNPDF40_an3lo_as_01180_mhou_t0119	(NNPDF40_an3lo_as_01180_qed_mhou_t0119)
NNPDF40_an3lo_as_01190_mhou_t0119	(NNPDF40_an3lo_as_01190_qed_mhou_t0119)
NNPDF40_an3lo_as_01200_mhou_t0119	(NNPDF40_an3lo_as_01200_qed_mhou_t0119)
NNPDF40_an3lo_as_01210_mhou_t0119	(NNPDF40_an3lo_as_01210_qed_mhou_t0119)
NNPDF40_an3lo_as_01220_mhou_t0119	(NNPDF40_an3lo_as_01220_qed_mhou_t0119)
NNPDF40_an3lo_as_01230_mhou_t0119	(NNPDF40_an3lo_as_01230_qed_mhou_t0119)
NNPDF40_an3lo_as_01240_mhou_t0119	(NNPDF40_an3lo_as_01240_qed_mhou_t0119)
NNPDF40_an3lo_as_01250_mhou_t0119	(NNPDF40_an3lo_as_01250_qed_mhou_t0119)

In addition, we also release sets in which multiplicative correlated uncertainties are determined in each case using the $t_0$ matrix corresponding to the respective value of $\alpha _s.$ Unlike the above sets, for these sets the replicas are not correlated across different values of $\alpha _s.$ These should not be used for $\alpha _s$ determination, for the reasons discussed in Sect. 3.1. However, if $\alpha _s$ is fixed as an external parameter they provide the most accurate prediction. They are denoted as

- NNLO QCD (+ QED effects)

**Table:** 

NNPDF40_nnlo_mhou_as_01140	(NNPDF40_nnlo_mhou_as_01140_qed)
NNPDF40_nnlo_mhou_as_01150	(NNPDF40_nnlo_mhou_as_01150_qed)
NNPDF40_nnlo_mhou_as_01160	(NNPDF40_nnlo_mhou_as_01160_qed)
NNPDF40_nnlo_mhou_as_01170	(NNPDF40_nnlo_mhou_as_01170_qed)
NNPDF40_nnlo_mhou_as_01180	(NNPDF40_nnlo_mhou_as_01180_qed)
NNPDF40_nnlo_mhou_as_01190	(NNPDF40_nnlo_mhou_as_01190_qed)
NNPDF40_nnlo_mhou_as_01200	(NNPDF40_nnlo_mhou_as_01200_qed)
NNPDF40_nnlo_mhou_as_01210	(NNPDF40_nnlo_mhou_as_01210_qed)
NNPDF40_nnlo_mhou_as_01220	(NNPDF40_nnlo_mhou_as_01220_qed)
NNPDF40_nnlo_mhou_as_01230	(NNPDF40_nnlo_mhou_as_01230_qed)
NNPDF40_nnlo_mhou_as_01240	(NNPDF40_nnlo_mhou_as_01240_qed)
NNPDF40_nnlo_mhou_as_01250	(NNPDF40_nnlo_mhou_as_01250_qed)
- ${\textrm{aN}}^3$LO QCD (+ QED effects)

**Table:** 

NNPDF40_an3lo_mhou_as_01140	(NNPDF40_an3lo_mhou_as_01140_qed)
NNPDF40_an3lo_mhou_as_01150	(NNPDF40_an3lo_mhou_as_01150_qed)
NNPDF40_an3lo_mhou_as_01160	(NNPDF40_an3lo_mhou_as_01160_qed)
NNPDF40_an3lo_mhou_as_01170	(NNPDF40_an3lo_mhou_as_01170_qed)
NNPDF40_an3lo_mhou_as_01180	(NNPDF40_an3lo_mhou_as_01180_qed)
NNPDF40_an3lo_mhou_as_01190	(NNPDF40_an3lo_mhou_as_01190_qed)
NNPDF40_an3lo_mhou_as_01200	(NNPDF40_an3lo_mhou_as_01200_qed)
NNPDF40_an3lo_mhou_as_01210	(NNPDF40_an3lo_mhou_as_01210_qed)
NNPDF40_an3lo_mhou_as_01220	(NNPDF40_an3lo_mhou_as_01220_qed)
NNPDF40_an3lo_mhou_as_01230	(NNPDF40_an3lo_mhou_as_01230_qed)
NNPDF40_an3lo_mhou_as_01240	(NNPDF40_an3lo_mhou_as_01240_qed)
NNPDF40_an3lo_mhou_as_01250	(NNPDF40_an3lo_mhou_as_01250_qed)

## Data Availability

This manuscript has associated data in a data repository. [Author’s comment: The datasets generated and/or analysed during the current study are available in the NNPDF repository (https://github.com/NNPDF/nnpdf).]

## Footnotes

1 A recent extension of the CMS analysis of [58] combines their inclusive jet production data at 2.76, 7, 8, and 13 TeV with inclusive HERA structure functions to yield $\alpha _s(m_Z)=0.1176_{-0.0016}^{+0.0014}$ [60].

## Code Availability Statement

This manuscript has associated code/software in a data repository. [Author’s comment: The code/software generated and/or analysed during the current study is available in the NNPDF repository (https://github.com/NNPDF/nnpdf).]
